# Neural control of immune cell trafficking

**DOI:** 10.1084/jem.20211604

**Published:** 2022-02-23

**Authors:** Scott N. Mueller

**Affiliations:** 1 Department of Microbiology and Immunology, The Peter Doherty Institute for Infection and Immunity, The University of Melbourne, Melbourne, Australia

## Abstract

Leukocyte trafficking between blood and tissues is an essential function of the immune system that facilitates humoral and cellular immune responses. Within tissues, leukocytes perform surveillance and effector functions via cell motility and migration toward sites of tissue damage, infection, or inflammation. Neurotransmitters that are produced by the nervous system influence leukocyte trafficking around the body and the interstitial migration of immune cells in tissues. Neural regulation of leukocyte dynamics is influenced by circadian rhythms and altered by stress and disease. This review examines current knowledge of neuro–immune interactions that regulate leukocyte migration and consequences for protective immunity against infections and cancer.

## Teaser

Cross-talk between the nervous system and the immune system is important in normal physiology and disease. This review discusses how neural signals regulate leukocyte migration around the body and within tissues and contributions by diverse neural signals to effective immune responses.

## Introduction

With the exception of subsets of immune cells that permanently reside in peripheral tissue niches, most mature leukocytes spend much of their cellular lives trafficking around the body with the goal of identifying and eradicating microorganisms and malignant cells. Soluble and cell-associated signals direct leukocyte trafficking, tissue entry, and migration within tissues. These include chemokines, cytokines, and adhesion molecules expressed by vasculature, tissues, and other immune cells (Nourshargh and Alon, 2014). In addition to these well-defined signals, neurotransmitters produced by the nervous system and adrenal glands can also impact leukocyte migration and functions. Both leukocyte-intrinsic and -extrinsic pathways are influenced by neural signals to direct migration. Investigation of the effects of neurotransmitters on leukocytes in the blood, in particular the catecholamines noradrenaline (also known as norepinephrine) and adrenaline (epinephrine), spans more than a century, providing clear evidence of their impact on systemic leukocyte trafficking. More recent studies have begun to elucidate interactions between the nervous system and the immune system within peripheral tissues and microanatomic niches, demonstrating the importance of location and the context of cellular differentiation on the outcome of neuro-immune communication. One area of particular interest is localized neural regulation of cell migration and contributions to homeostasis and disease. Modulation of neuro–immune communication in tissues using targeted therapies is an exciting goal that could have an extraordinary impact on disease treatments, but we still have much to learn. This review discusses how neural pathways that influence immune cell trafficking during homeostasis and stress potentially shape the outcomes of immune responses to diverse diseases.

## A brief overview of neural pathways relevant to the immune system

**Figure 1.**
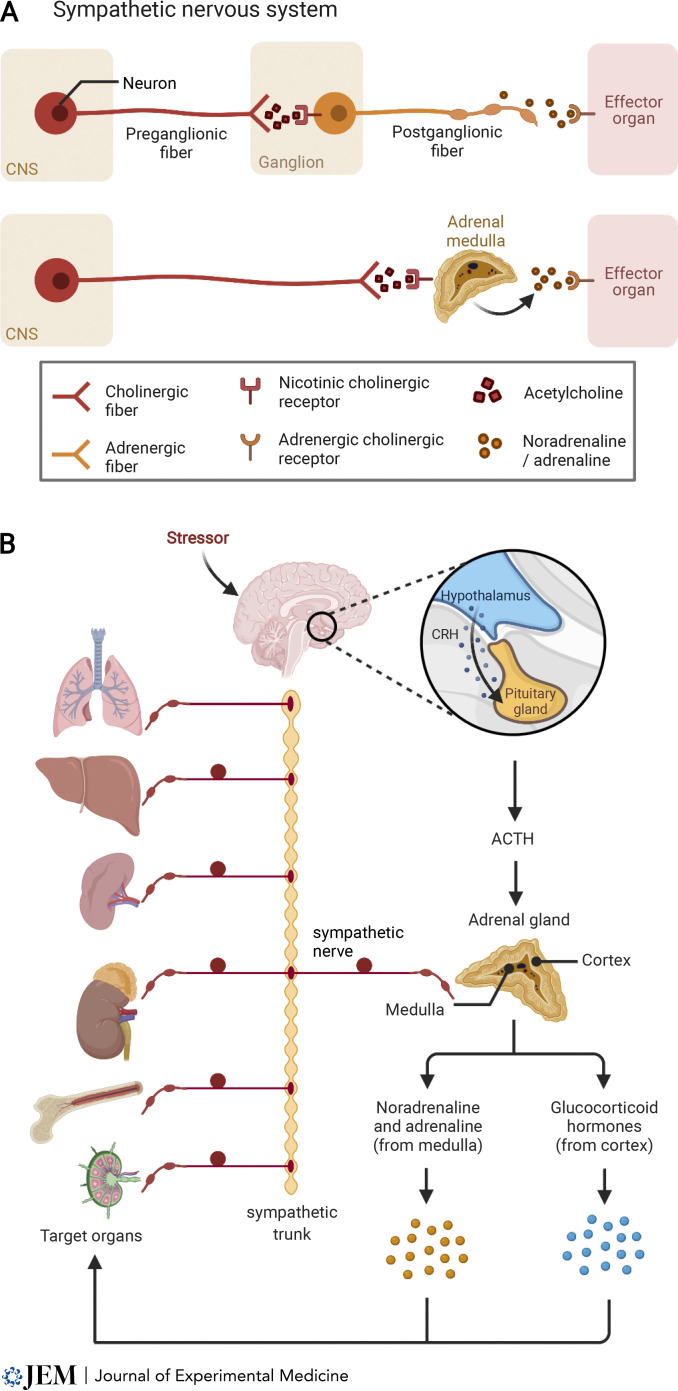
**Schematic of the organization of the SNS. (A)** Neurons project from the central nervous system (CNS) to ganglia and to the adrenal medulla, where these preganglionic nerves communicate by releasing acetylcholine. Activation of postganglionic nerves leads to noradrenaline release in target organs. Sympathetic stimulation of the adrenal medulla leads to release of adrenaline and noradrenaline. **(B)** Stress activates sympathetic nerves that release noradrenaline in target organs (examples shown here: lungs, liver, spleen, kidney with adrenal gland, bone, and LN). Circles above sympathetic nerves are diagrammatic representations of sympathetic ganglia, and beads represent varicosities. Activation of the HPA axis increases corticotropin-releasing hormone (CRH) that induces adrenocorticotropic hormone (ACTH) release into the blood from the pituitary gland. ACTH triggers adrenaline and noradrenaline secretion from the adrenal medulla and cortisol production from the adrenal cortex that can act systemically on target organs.

The nervous system is composed of a somatic arm that consists of efferent motor nerves and afferent sensory nerves, and an autonomic branch that consists of parasympathetic and sympathetic nerves. Sensory nerves comprise at least five functional classes, including nociceptors that sense pain and noxious stimuli and signal via neurotransmitters, including the neuropeptides calcitonin gene-related peptide (CGRP) and substance P (Usoskin et al., 2015). Motor neurons, preganglionic (nerves that connect the central nervous system to the ganglia) and postganglionic (nerves that innervate target tissues) parasympathetic nerves, and preganglionic sympathetic nerves transmit their signals via the neurotransmitter acetylcholine (Fig. 1 A). In contrast, most postganglionic sympathetic nerves transmit signals via axonal release of noradrenaline, and in many cases also neuropeptide Y.

Most tissues in the body are innervated to varying degrees by both sensory and autonomic nerves. Pertinent to neuro-immune communication is the observation that primary (bone marrow and thymus) and secondary (spleen and LN) lymphoid organs are innervated by sympathetic nerves (Cleypool et al., 2021; Felten et al., 1984; Giron et al., 1980). In LNs, most innervation occurs in the hilum and medullary regions. In addition to paravascular sympathetic innervation of LN blood vessels, nerve fibers can penetrate into T cell zones and toward the border of the B cell follicles. In contrast, sensory innervation of LNs is limited to the capsule and hilum (Huang et al., 2021; Popper et al., 1988). In the spleen, sympathetic innervation is prominent around the central arteries, but fibers also branch into the T cell zones (Ding et al., 2019; Felten et al., 1985; Nance and Burns, 1989), where both T cells and macrophages or dendritic cells (DCs) have been described in close association with nerve terminals (Felten et al., 1987). This provides a theoretically rapid means of communication between the sympathetic nervous system (SNS) and leukocytes in lymphoid organs. Sympathetic nerves in target organs contain varicosities, which bear resemblance to beads on a string and are the sites from which transmitters are released in response to a nerve impulse. Thus, neurotransmitter release occurs at multiple sites along the axon rather than at a single axon synapse. Despite these long-standing observations, direct demonstration of signaling events between sympathetic nerves and local immune cells in tissues is currently lacking.

In stark contrast to most other organs, there is no parasympathetic innervation in lymphoid organs, indicating that adrenergic signaling is a principal means of communication between the nervous system and the immune system (Nance and Sanders, 2007). The two arms of the autonomic nervous system typically act to balance arousal (sympathetic: “fight or flight”) with homeostasis (parasympathetic: “rest and digest”). However, under inflammatory conditions, lymphocytes have been shown to express choline acetyltransferase and produce acetylcholine (Cox et al., 2019; Olofsson et al., 2016; Reardon et al., 2013), which provides a non-neuronal pathway for regulation of diverse processes including blood pressure and macrophage functions (Olofsson et al., 2016; Rosas-Ballina et al., 2011). Similarly, myeloid cells can express tyrosine hydroxylase, the rate-limiting enzyme responsible for catecholamine biosynthesis (Flierl et al., 2007). It was shown that myeloid-derived catecholamines amplify inflammation induced in response to systemic infection or endotoxin (Staedtke et al., 2018). Further studies are needed to unravel the role of neurotransmitters produced by leukocytes.

The adrenal medulla is the source of systemic adrenaline and little circulating noradrenaline. Most noradrenaline in blood comes from spillover from sympathetic nerve terminals (Goldstein et al., 1983). The adrenal medulla is the central part of the adrenal glands, which are located above the kidneys. The adrenal cortex secretes steroid hormones including glucocorticoids (e.g., cortisol) and mineralocorticoids. In response to acute psychological stress, activation of the hypothalamic-pituitary-adrenal (HPA) axis drives sympathetic preganglionic neurons that stimulate chromaffin cells in the adrenal medulla (Fig. 1 B). Activation of the HPA axis, including sustained activation, also leads to glucocorticoid production by the adrenal cortex. Cytokines produced during infection can activate the HPA axis and induce glucocorticoids that ultimately contribute to control of inflammation (Miller et al., 1997). These steroid hormones have multiple roles in regulating leukocyte functions, as discussed in detail by others (Cain and Cidlowski, 2017; Liberman et al., 2018).

## Adrenergic regulation of immune responses: A complex balancing act

The impact of the SNS on the immune system remains an area of intense interest. Catecholaminergic signaling occurs through G-protein–coupled α- and β-adrenergic receptors. The three main subtypes include α_1_-adrenoceptor subtypes (α_1_A, α_1_B, and α_1_D), α_2_ subtypes (α_2_A, α_2_B, and α_2_C), and β-adrenoceptors (β_1_, β_2_, and β_3_) that are expressed on the surface of diverse cell types. α_1_-Adrenoceptors are expressed on smooth muscle cells and control vasoconstriction in blood vessels. α_2_-Adrenoceptors are involved in vasoconstriction (α_2_B) and presynaptic inhibition of noradrenaline release (α2A/C). The β-adrenoceptors also play diverse functional roles, including increased cardiac output (β_1_) and smooth muscle relaxation leading to dilatation of the airways (β_2_). Myeloid cells express both α- and β-adrenoceptors, whereas lymphocytes express mostly β_2_-adrenoceptors.

β-Adrenoceptor signaling is often associated with suppression of immune responses by reducing or impairing immune functions such as inflammatory cytokine production (Elenkov et al., 2000; Kohm and Sanders, 2001; Madden et al., 1995; Severn et al., 1992). β_2_-Adrenoceptor signaling induces intracellular increases in cAMP that activate protein kinase A, leading to transcriptional regulation of diverse pathways (Lorton and Bellinger, 2015). However, suppression is not the default outcome of adrenoceptor signaling, as there are several examples of increased immune function following adrenergic signaling, such as increased inflammation in response to α-adrenoceptor stimulation of macrophages (Nakai and Suzuki, 2019; Pongratz et al., 2014; Sanders, 2012).

Early in vitro studies found that stimulation of naive T cells with noradrenaline or β-adrenoceptor agonists during priming increased T cell cytokine production, whereas stimulation of effector cells generally inhibited T cell proliferation or reduced cytokine production (Estrada et al., 2016; Madden et al., 1995; Sanders, 2012; Swanson et al., 2001). Similarly, noradrenaline was shown to enhance B cell responses when present at priming but did not improve responses when administered after priming (Kouassi et al., 1988; Sanders and Munson, 1984). In contrast, β_2_-adrenoceptor signaling in CD4^+^ T regulatory cells can increase suppressive activity that might contribute to suppression of effector responses. Experiments using chemical sympathectomy with 6-hydroxydopamine before induction of T cell responses supports experiments with catecholamines by reducing CD8 T cell priming in some models (Leo et al., 1998; Livnat et al., 1987); but loss of sympathetic innervation led to increased responses in other models (Grebe et al., 2009; Madden et al., 1994).

Integrating the information from these and many other studies performed over the past few decades (Kohm and Sanders, 2001; Nance and Sanders, 2007; Sanders, 2012) reveals that the context of adrenergic signaling is paramount to the resulting impact on immune responses. The outcomes of adrenergic signaling are influenced by the differentiation status of the cell (presumptively due to changes in the relative expression and activation of different signaling pathways within cells), the subtype of adrenoceptors expressed by cells, and the level of expression of these and other receptors. Coexpression of different adrenoceptors also provides a subtle mechanism for fine-tuning of signaling in cells. α-Adrenoceptors have a higher affinity for noradrenaline compared with adrenaline, whereas β-adrenoceptors have a higher affinity for adrenaline (Molinoff, 1984). Consequently, α-adrenergic signaling may dominate when concentrations of noradrenaline are low, but at higher concentrations, β-adrenoceptor signals will dominate. This has been demonstrated in macrophages responding to LPS (Pongratz and Straub, 2014), but it remains to be determined if specific functions of immune cells are regulated as a function of distance from nerve terminals in tissues and neurotransmitter concentration, especially lymphocytes that express almost exclusively β2-adrenoceptors. Therefore, the magnitude of sympathetic activity and availability of neurotransmitters in tissues will influence responses. Increased sympathetic nerve activity and increased nerve density have been demonstrated following periods of behavioral stress and may alter the outcomes of immune responses (Sloan et al., 2008a; Sloan et al., 2007).

In sum, despite the large number of studies investigating adrenoceptor signaling in leukocytes, we still need to precisely define how the differentiation state of immune cells influences the outcomes of both cell intrinsic and extrinsic adrenoceptor signaling on cellular functions such as migration.

## Regulation of homeostatic leukocyte trafficking by the nervous system

Many leukocytes spend considerable time trafficking in the blood. Neutrophils and monocytes spend the majority of their short lives in the circulation (Patel et al., 2021). Conversely, lymphocytes continually migrate from blood into secondary lymphoid organs and back again via the lymphatics, spending on average 12–21 h in LNs, where they scan for cognate antigen by interacting with APCs (Mandl et al., 2012). Migration is controlled by expression of adhesion molecules and chemokine receptors on leukocytes and expression of reciprocal ligands by endothelial cells that construct the blood and lymphatic vessels that serve as tissue entry and exit points for leukocytes. As discussed below, recent studies have demonstrated that regulation of cell trafficking under homeostatic conditions is partially under control of circadian rhythms that are driven by neural and humoral signals (Leach and Suzuki, 2020).

### Neural regulation of cell egress from bone marrow

The suprachiasmatic nucleus in the hypothalamus is the central clock that generates circadian rhythms, which include diurnal regulation of the HPA axis and adrenal glucocorticoid production, as well as diurnal fluctuations in sympathetic nerve activity. This results in an increase in noradrenaline and adrenaline in the circulation during the phase when animals are most active, during the day in humans and at night in mice. Circadian rhythms are regulated by the light–dark cycle. Elegant studies have demonstrated both leukocyte-intrinsic and -extrinsic circadian regulation of molecules involved in immune cell trafficking. A prime example of this is in the bone marrow, where circadian release of noradrenaline by sympathetic nerves in the bone marrow downregulates expression of CXCL12 in stromal cells via β_3_-adrenoceptor signaling (Mendez-Ferrer et al., 2008). Diurnal changes in CXCL12 expression lead to release of CXCR4-expressing hematopoietic stem cells. Cholinergic signals were shown to be important for controlling sympathetic noradrenergic tone in bone marrow in a circadian manner (Garcia-Garcia et al., 2019). This appears to be distinct from pharmacological G-CSF–induced noradrenaline release, which drives downregulation of CXCL12 in osteoblasts via β_2_-adrenoceptor signaling that mobilizes hematopoietic stem cells (Katayama et al., 2006). Because G-CSF is produced after infection, these observations suggests that distinct adrenergic-mediated pathways converge to control cell egress from the bone marrow niche in homeostasis and disease. Moreover, the homeostatic control of hematopoietic stem cell release from the bone marrow is also controlled by sensory nerves (Gao et al., 2021). It was found that the sensory neurotransmitter CGRP promotes hematopoietic stem cell mobilization via calcitonin receptor-like receptor (CALCRL) and receptor activity modifying protein 1 (RAMP1), potentially via G-CSF–mediated release of CGRP from nociceptors. Hence, G-CSF appears to stimulate sympathetic and sensory nerves in the bone marrow. This is an intriguing example of sensory, noradrenergic, and cholinergic neural signals together controlling leukocyte dynamics, which might additionally be enhanced in response to inflammation to promote hematopoiesis (Suekane et al., 2019).

### Circadian regulation of promigratory pathways in leukocytes and tissues

**Figure 2.**
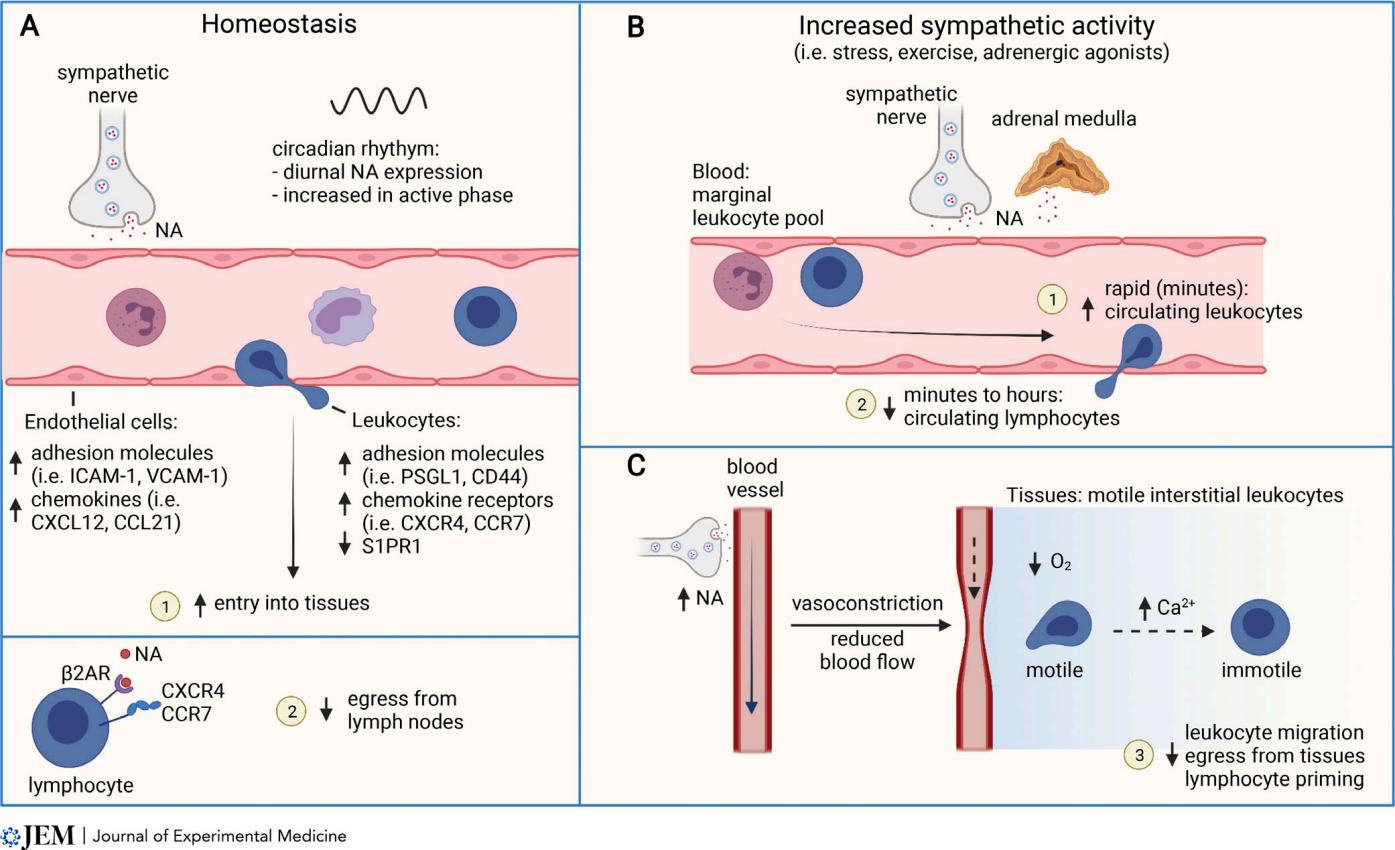
**Regulation of leukocyte migration and trafficking by the SNS. (A)** Homeostatic regulation of leukocyte trafficking is influenced by diurnal oscillations in SNS activity and increased noradrenaline release during the active phase (day in humans, night in mice). Endothelial cells and leukocytes increase expression of adhesion molecules and chemokines or chemokine receptors and promote leukocyte entry into tissues (1). In LNs, the circadian increase in noradrenaline stimulates reduced lymphocyte egress via β_2_-adrenoceptors (β_2_AR) and the chemokine receptors CXCR4 and CCR7 (2). **(B)** Increased sympathetic activity in response to stress, exercise, or pharmacological adrenergic agonists induces a rapid increase in leukocyte numbers in the blood within minutes (1), followed by a decrease in circulating lymphocyte numbers (2). **(C)** Increased sympathetic activity induces blood vessel constriction, reduced blood flow and tissue oxygenation. Leukocytes respond to hypoxia through calcium signaling that impairs cell motility, impacting tissue egress and cellular immunity (3).

Oscillations in cell trafficking are controlled by rhythmic changes in adhesion molecule expression by endothelial cells, including ICAM-1, VCAM-1, P- and E-selectin, and MAdCAM-1, which are required for extravasation of leukocytes from the circulation in the tissues (Fig. 2 A). Diurnal changes in expression of these molecules is controlled by local sympathetic nerves (He et al., 2018; Scheiermann et al., 2012). Notably, regulation of adhesion molecule expression varies by tissue and by leukocyte subset, potentially facilitating fine-grained control of cell trafficking. Moreover, diurnal regulation of adhesion molecules in blood endothelial cells by the SNS can differ between arteries and veins, leading to greater cell adhesion in arteries in the morning and veins in the evening (de Juan et al., 2019). Neutrophils, B cells, natural killer (NK) cells, monocytes, and eosinophils also migrate into tissues more at night in mice (He et al., 2018), and neutrophils migrate more into tissues in models of systemic inflammation and septic shock when administered to mice at night (Scheiermann et al., 2012). Interestingly, diurnal tissue entry might be further reinforced by increased adhesion to venules at night (de Juan et al., 2019; Scheiermann et al., 2012). Finally, since lymphatic vessel contractility in tissues is also regulated by the SNS (Bachmann et al., 2019), rhythmic control of lymph flow and leukocyte exit from tissues might also contribute to these fluctuations in cell trafficking.

Together with control of promigratory factors by tissues, chemokine receptor and integrin expression in leukocytes, including CXCR4, CD44, and CD11a, is also under circadian control (Fig. 2 A). Lymphocytes regulate the cell surface receptors CCR7 and S1PR1 in a circadian manner that enhances homing into LNs at night in mice (Druzd et al., 2017; He et al., 2018; Pelegri et al., 2003). Rhythmic receptor expression in lymphocytes requires the clock genes, and loss of the core clock gene Bmal1 in T cells abrogates circadian T cell homing to LNs (Druzd et al., 2017). Despite these effects, genetic deletion of clock genes does not appear to markedly impact T cell responses to immunization or infection (Hemmers and Rudensky, 2015). Higher levels of noradrenaline in LNs in mice at night also act to reduce the egress of lymphocytes in a manner that requires β_2_-adrenoceptor expression on lymphocytes (Suzuki et al., 2016). Mechanistically, β_2_-adrenoceptors were found to interact with the chemokine receptors CCR7 and CXCR4 (Fig. 2 A). The β_2_-adrenoceptor–mediated reduction in LN egress was mediated primarily by CXCR4 in T cells and CCR7 in B cells (Nakai et al., 2014).

### Neural control of diurnal leukocyte trafficking influences immune responses

Circadian control of leukocyte recruitment and egress from lymphoid tissues was found to impact the induction of humoral immune responses after protein immunization with the hapten 4-hydroxy-3-nitrophenyl acetyl conjugated with chicken γ-globulin protein (Suzuki et al., 2016). An increase in antibody titers, germinal center B cells, and CD4^+^ T follicular helper cell numbers was observed when mice were immunized at night, which was dependent on sympathetic innervation and reduced in mice lacking the β_2_-adrenoceptor. This enhanced immunity persisted for ≥14 d, which is remarkable considering that T and B cell priming occurs over a period of days to weeks and circadian regulation is diurnal. However, examination of the role of clock genes in CD8^+^ T cells responding to immunization with peptide-loaded DCs revealed an influence on the early priming of T cells (Nobis et al., 2019), indicative of reduced encounters between naive T cells and APCs. Hence, circadian control of homeostatic leukocyte migration, via cell intrinsic and extrinsic pathways that are influenced by oscillatory SNS activity, can influence the magnitude of immune responses.

The circadian influence on immune responses could have important implications for vaccination or therapies, whereby timing delivery to the peak of tissue lymphocyte numbers in patients (morning) may improve immunity. Indeed, a recent study directly examined this in volunteers receiving the tuberculosis vaccine bacillus Calmette-Guérin (BCG; de Bree et al., 2020). Morning vaccination resulted in improved cytokine production by peripheral blood leukocytes after restimulation. However, immune responses to viral and bacterial infection models in mice were found to be largely insensitive to loss of the circadian clock in lymphocytes (Hemmers and Rudensky, 2015). Yet the circadian clock also regulates the homeostasis and functions of many innate immune cells that shape the outcomes of immune responses (Palomino-Segura and Hidalgo, 2021). Increased antigen availability or more protracted antigen presentation during infection compared with vaccination would reduce the risk that exposure to a pathogen in the rest phase will diminish protective adaptive immunity. Hence, the evolutionary significance of neurally driven, circadian regulation of leukocyte trafficking may be to increase cell recruitment to tissues when animals are more active and have a greater likelihood of injury or exposure to disease in barrier tissues. It will be important for future studies to determine if subsets of naive and effector or memory T cells are differentially directed to tissue niches in response to rhythmic sympathetic activity, for instance, by promoting naive lymphocyte recruitment to lymphoid tissues to increase the likelihood of encountering cognate antigens, versus directing antigen-experienced lymphocytes to nonlymphoid tissues, where they could rapidly respond to pathogens.

## Modulation of leukocyte trafficking in the circulation by stress-induced catecholamines and hormones

### Catecholamines

Increased catecholamine availability during stress or following pharmacological stimulation of adrenergic signaling also redirects the trafficking of leukocytes in the circulation (Fig. 2 B). Neuronal activation of the adrenal gland induces the release of adrenaline, and to a lesser extent noradrenaline, into the circulation, which has systemic effects on the organism. Soon after the discovery and isolation of adrenaline, it was observed that its injection into animals induces a rapid leukocytosis (reviewed in Benschop et al., 1996). Summarizing numerous studies of the injection of adrenaline or noradrenaline into animals and humans reveals two phases of catecholamine-induced changes in leukocyte frequency in the blood: a rapid increase in lymphocytes (peaking within 30 min) is followed by an increase in neutrophils and monocytes that peaks after 2–4 h, and a subsequent decrease in lymphocytes. Leukocytosis is more pronounced in response to injected adrenaline compared to noradrenaline (Dhabhar et al., 2012). This response is also adrenoceptor subtype dependent, whereby β_2_-adrenoceptor agonists mostly increase circulating NK cells and CD8^+^ T lymphocytes, while α-adrenoceptor stimulation induces an increase in neutrophils (Benschop et al., 1996; Gader and Cash, 1975).

The rapid release of leukocyte subsets into the circulation following adrenergic stimulation is assumed to result in the release of cells from so-called marginal pools and highly vascularized tissues such as the spleen and lungs. Indeed, within 5 min of injection of noradrenaline or the nonselective β-adrenoceptor agonist isoprenaline, leukocyte release from the spleen increases (Ernstrom and Sandberg, 1973). Injection of LPS or IL-1 also increases spleen blood flow and cell exit (Rogausch et al., 1995; Rogausch et al., 1997; Rogausch et al., 1999). Exercise induces the release of T cells from the spleen via adrenergic signaling (Kruger et al., 2008). However, after the initial increased release of leukocytes from the spleen in response to noradrenaline, spleen blood flow slows and output of lymphocytes from the spleen is reduced via α-adrenoceptor–dependent signaling (Rogausch et al., 1999). Importantly, the basal release of cells from the spleen was not found to differ after sympathectomy (Ernstrom and Sandberg, 1973), implying that homeostatic regulation of leukocyte egress from the spleen is either not under sympathetic control or could not be detected in these experiments.

### Glucocorticoids

Glucocorticoid hormones also induce changes in leukocyte trafficking. Injection of patients with hydrocortisone reduces the numbers of lymphocytes and monocytes in the blood after 4–6 h (Fauci and Dale, 1974) coinciding with an increase in the ability of T and B cell to home to the bone marrow (Fauci, 1975). Likewise, the circadian increase in cortisol in the morning in humans increases expression of CXCR4 and IL-7Rα on T cells, but does not alter expression of CCR7 or CD62L, and reduces T cells in the blood (Besedovsky et al., 2014a; Besedovsky et al., 2014b; Shimba et al., 2018). Reorganization of cellular cortical actin reduces cell stiffness in response to glucocorticoids and catecholamines (Fay et al., 2016; Kim et al., 2019b), which may explain rapid leukocytosis via demargination of cells that become softer and “fall” into circulation.

### Stress-induced changes in leukocyte trafficking in blood

The changes in blood leukocyte numbers in response to injected catecholamines or glucocorticoids reflect changes induced in response to models of stress in rodents (Dhabhar et al., 2012). Lymphocyte numbers increase in the blood within 6 min following restraint stress in rats, then begin decreasing in number for ≥2 h. In contrast, neutrophil numbers increase and remain high in the blood for ≥2 h after stress. Stress associated with major surgery is also correlated with increased catecholamines during surgery and increased cortisol that is sustained postsurgery (Ogawa et al., 2000). Surgery induces a redistribution of lymphocytes from the blood to LNs (Toft et al., 1993) that can persist for weeks after surgery (Ogawa et al., 2000). Other psychological and physiological stressors that increase circulating catecholamines or glucocorticoids likely have similar impacts on leukocyte trafficking.

Together, these numerous observations suggest that activation of the HPA axis in response to stress leads to an increase in circulating adrenaline and glucocorticoids, mostly from endocrine tissues, that first mobilizes cells into circulation from marginal vascular pools and subsequently increases cell trafficking into tissues, where production of noradrenaline by local sympathetic nerves has an additional influence on cell trafficking and cell behavior (see the next section; Fig. 2). Although the predominant tissue destinations of altered cell trafficking in responses to stress-induced catecholamines and glucocorticoids are poorly defined, inflamed tissues are a presumptive target where the body may benefit from directing cells in response to an acute stressor to respond to tissue damage or infection. In support of this, stress can increase leukocyte migration into the surgical site (Viswanathan and Dhabhar, 2005), where leukocytes are required to help restore tissue homeostasis.

## Neural control of dynamic leukocyte motility in tissues

Upon entering tissues from the blood, leukocytes move with dynamic motility and navigate diverse microenvironments. Motility is essential for leukocyte functions in tissues. Cells must adapt to various physical, cellular, extracellular matrix, and soluble cues within tissues (Fowell and Kim, 2021). Interstitial motility facilitates directional recruitment of leukocytes toward sites of infection or tissue injury and enables random interactions between APCs and lymphocytes searching for foreign antigens or infected cells. Leukocytes integrate chemotactic and adhesive cues that facilitate rapid locomotion through intracellular actin dynamics that polarize the cell and generate force that propels the cell forward (Lammermann and Sixt, 2009). Signals that influence the actin cytoskeleton can substantially alter or even halt leukocyte motility. A well-documented example is T cell arrest in response to TCR stimulation. TCR signaling results in increased intracellular calcium that impacts actinomyosin-driven motility (Negulescu et al., 1996).

Recent work has shown that catecholamines can also substantially regulate leukocyte motility in tissues (Devi et al., 2021; Fig. 2 C). By using intravital multiphoton imaging to follow leukocyte movement in tissues in live mice, activation of the SNS was found to impair cell migration. Both systemic and local noradrenaline, as well as chemogenetic activation of sympathetic nerves, rapidly suppress the motile behavior of leukocytes in diverse tissues including the LNs, skin, and liver. Catecholamines can impact the migration of naive T and B lymphocytes, memory T cells, and DCs. The increased production of noradrenaline following stroke was also found to impair the motility of NKT cells in the liver (Wong et al., 2011). Mechanistically, adrenergic signaling in nonhematopoietic cells induces vasoconstriction in peripheral blood vessels, which swiftly impacts tissue oxygenation (Devi et al., 2021). Regulation of blood flow and oxygen availability in tissues by the SNS results in a rapid increase in intracellular calcium that coincides with impaired cell motility within minutes (Devi et al., 2021). Reduced oxygen tension in tissues also correlates with reduced T cell migration speed (Rytelewski et al., 2019). The modulation of cell migration by catecholamines acts via the vasculature and therefore does not require expression of adrenergic receptors by leukocytes and can be reversed by increasing tissue oxygenation (Devi et al., 2021). Importantly, sympathectomy by 6-hydroxydopamine does not alter basal leukocyte motility, supporting the conclusion that control of leukocyte migration requires increased activation of sympathetic nerves, leading to vasoconstriction.

These observations illustrate that acute stress-induced release of catecholamines can rapidly restrict the motility of leukocytes. This pause in leukocyte behavior was short-lived in healthy animals but was sustained for hours in mice with a viral infection (Devi et al., 2021). Restricting leukocyte motility might control the energy requirements of migrating leukocytes (Marelli-Berg and Jangani, 2018). During an acute systemic threat, controlling leukocyte migration may help to conserve resources that are required by tissues including the muscles, lungs, and heart to escape danger. However, restricting cell motility in the LNs during the priming phase of viral or parasitic infection, or in response to tumors, suppressed the induction of T cell responses (Devi et al., 2021). The impact of neuronal activation on immune cells will depend on the timing, magnitude, and duration of sympathetic activity but will presumably also be impacted by the health of the individual. Underlying health conditions that intensify or compound sympathetic activity would be expected to increase the impact on leukocytes. For instance, peripheral vasoconstriction is increased in patients with heart disease (Kim et al., 2019a). Conversely, studies have shown that loss of sympathetic innervation can occur in lymphoid organs during simian immunodeficiency virus infection and in patients with end-stage sepsis (Hoover et al., 2017; Sloan et al., 2008b). Clearly, further insights into the role of sympathetic neuro-immune communication in tissues are required to fully understand the impact on immune responses.

## Neural control of leukocyte recruitment to tissues

### Sympathetic nerves

In addition to the systemic control of leukocyte trafficking by catecholamines in the circulation, the innervation of peripheral tissues by sympathetic and sensory nerves provides many avenues for locally confined neuro-immune interactions. Clear examples of such interactions include the demonstration that muscularis macrophages in the intestine upregulate tissue repair genes, including arginase-1 via β_2_-adrenoceptor stimulation in response to noradrenaline produced by local sympathetic nerves (Gabanyi et al., 2016). Optogenetic activation of sympathetic nerves in the colon was found to reduce expression of MAdCAM1 and ICAM1 on endothelial cells via the β_2_-adrenoceptor, thereby reducing leukocyte recruitment to the colon and ameliorating experimental colitis (Schiller et al., 2021).

### Sensory nerves

**Figure 3.**
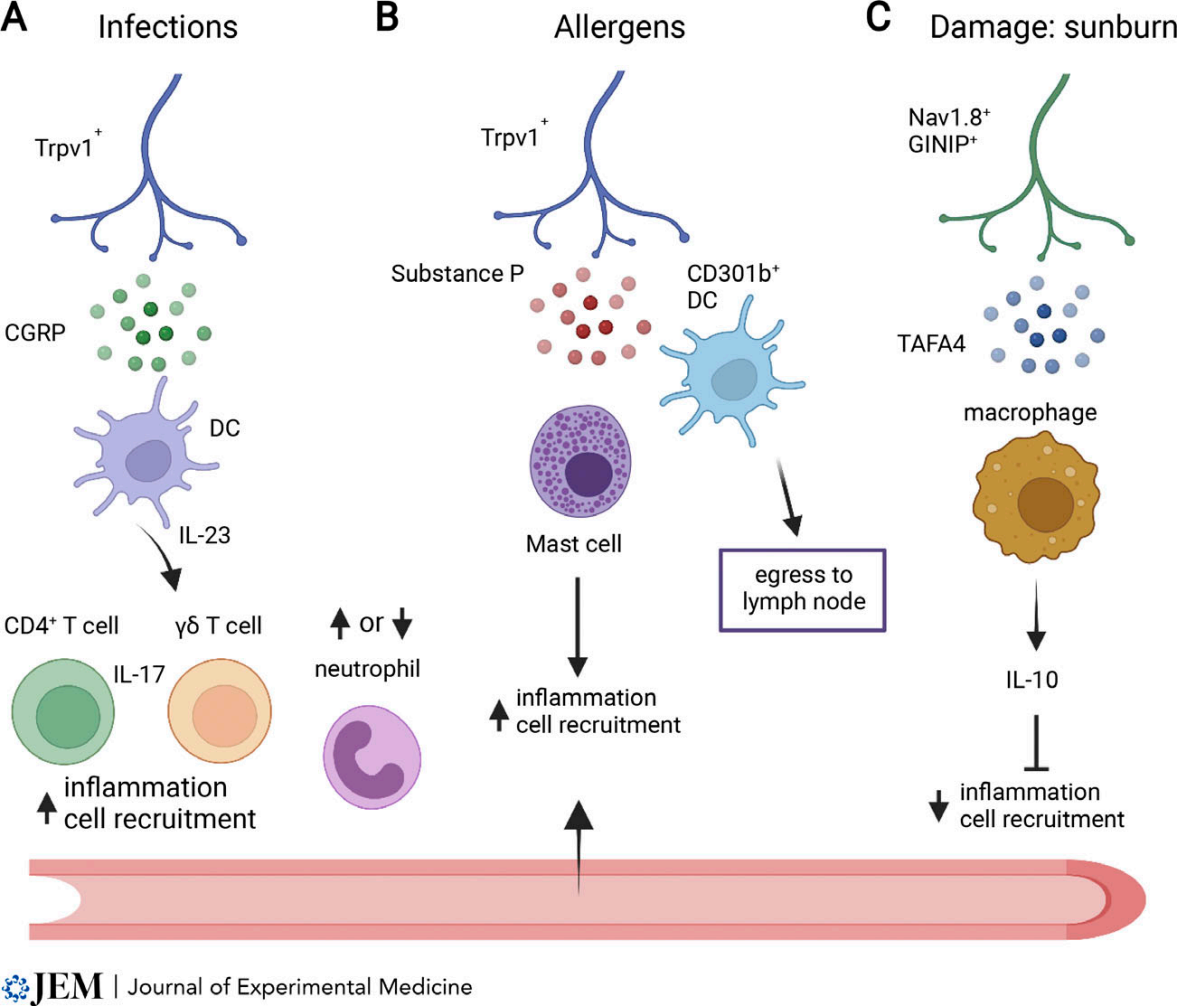
**Regulation of leukocyte recruitment to tissues by sensory nerves. (A)** Trpv1^+^ nociceptors produce CGRP in response to infection or inflammation that can act on DCs and induce IL-23, which induces inflammatory recruitment of IL-17–producing CD4^+^ T cells, γδ T cells, and neutrophils. CGRP can also suppress recruitment of neutrophils in the lung during bacterial infection. **(B)** Production of substance P by Trpv1^+^ nociceptors in response to allergens can induce migration of dermal CD301b^+^ DCs to LNs via Mrgpra1 and activate mast cells via Mrgprb2 to increase local inflammation. **(C)** Nonpeptidergic Nav1.8^+^ GINIP^+^ nociceptors produce TAFA4 in response to damage induced by sunburn and increase IL-10 production by macrophages that can dampen inflammation and cell recruitment to tissues.

The activity of sensory nerves can also shape leukocyte responses in tissues niches. In the skin, macrophages and DCs interact with Trpv1^+^ sensory nerves in the dermis (Kolter et al., 2019), and sensory nerves are required to stimulate IL-23 production by dermal DCs following stimulation by the TLR7 agonist imiquimod (Riol-Blanco et al., 2014). Optogenetic activation of Nav1.8^+^ Trpv1^+^ nerves leads to CGRP release both locally and in adjacent skin through axon reflex activation of local Trpv1^+^ nerves. This was found to enhance local inflammation and to recruit IL-17^+^ γδT and CD4^+^ T cells (Fig. 3 A), providing protection against a subsequent infection adjacent to the site of optogenetic sensory nerve activation (Cohen et al., 2019). Conversely, allergens activate Trpv1^+^ neurons in the skin, which release substance P and act via Mrgpra1 to drive the migration of CD301b^+^ DCs to draining LNs (Perner et al., 2020), and can stimulate MrgprB2 expressed on mast cells to promote local inflammation that recruits immune cells (Green et al., 2019; Fig. 3 B). Substance P has also been found to be chemotactic for lymphocytes in vitro (Schratzberger et al., 1997). Another distinct response by skin sensory nerves involves Nav1.8^+^ GINIP^+^ neurons that produce the neuropeptide TAFA4 in response to UV skin damage (Hoeffel et al., 2021). This response promotes IL-10 production by dermal macrophages, reducing inflammation and cell infiltration (Fig. 3 C).

Trpv1^+^ nociceptors were also found to release CGRP and inhibit neutrophil recruitment, intravascular crawling behavior in the lung, and accumulation of lung γδ T cells during bacterial infection (Baral et al., 2018), as well as neutrophil recruitment during *Streptococcus pyogenes* infection (Pinho-Ribeiro et al., 2018). Sensory neurons can also express inflammatory chemokines and cytokines in peripheral ganglia in response to TLR signaling, which contribute to recruitment of macrophages (Liu et al., 2016). Finally, the neurotransmitter serotonin (5-hydroxytryptamine) has been shown to have multiple effects on immune cells (Hodo et al., 2020), including stimulating DC migration (Muller et al., 2009). These specific examples serve to highlight the potential for localized neuro-immune communication in defined tissues, driven by external stimuli and reflex neural circuits (Andersson and Tracey, 2012).

## Neural regulation of leukocyte migration in cancer

Recent studies have also revealed that peripheral nerves can influence tumor growth and metastasis (Zahalka and Frenette, 2020), highlighting potential avenues for neural-targeted treatments that augment current therapies, including immunotherapies. To achieve this, a better understanding of tumor innervation is required (Monje et al., 2020), as well as defining the balance of positive and negative effects of neural signaling on leukocytes and tumor cells. Preclinical studies have shown that sympathetic nerves can directly enhance tumor progression (Kamiya et al., 2019). Innervation of tumors by the SNS contributes to vascular remodeling in the tumor microenvironment to support metastasis (Le et al., 2016; Renz et al., 2018; Thaker et al., 2006), which might conceivably also impact leukocyte trafficking. Neural signaling induced by physical restraint stress or cold stress increased recruitment of M2 macrophages and myeloid-derived suppressor cells into the tumor microenvironment (Mohammadpour et al., 2019; Sloan et al., 2010) and reduced numbers of CD8^+^ T cells in tumors (Kokolus et al., 2013). However, the mechanisms of neural control of leukocyte trafficking to tumors remain unclear. The impact of catecholamines on leukocyte migration in tissues via vasoconstriction (Devi et al., 2021) suggests that the impact of stress-induced increases in adrenergic signaling within tumors might increase already dysfunctional blood flow and tumor hypoxia. This would be predicted to reduce the recruitment of immune cells into the tumor microenvironment and contribute to impaired antitumor immunity. However, evidence for neural control of leukocyte motility in the tumor microenvironment is lacking. Nevertheless, early clinical trials using β-blockers indicate that blocking adrenergic pathways can reduce metastasis and improve the recruitment of immune cells into tumors (Galluzzi et al., 2020; Hiller et al., 2020; Kokolus et al., 2018). It is clear that further investigation of these pathways could reveal opportunities to improve cancer treatments.

## Final thoughts

**Figure 4.**
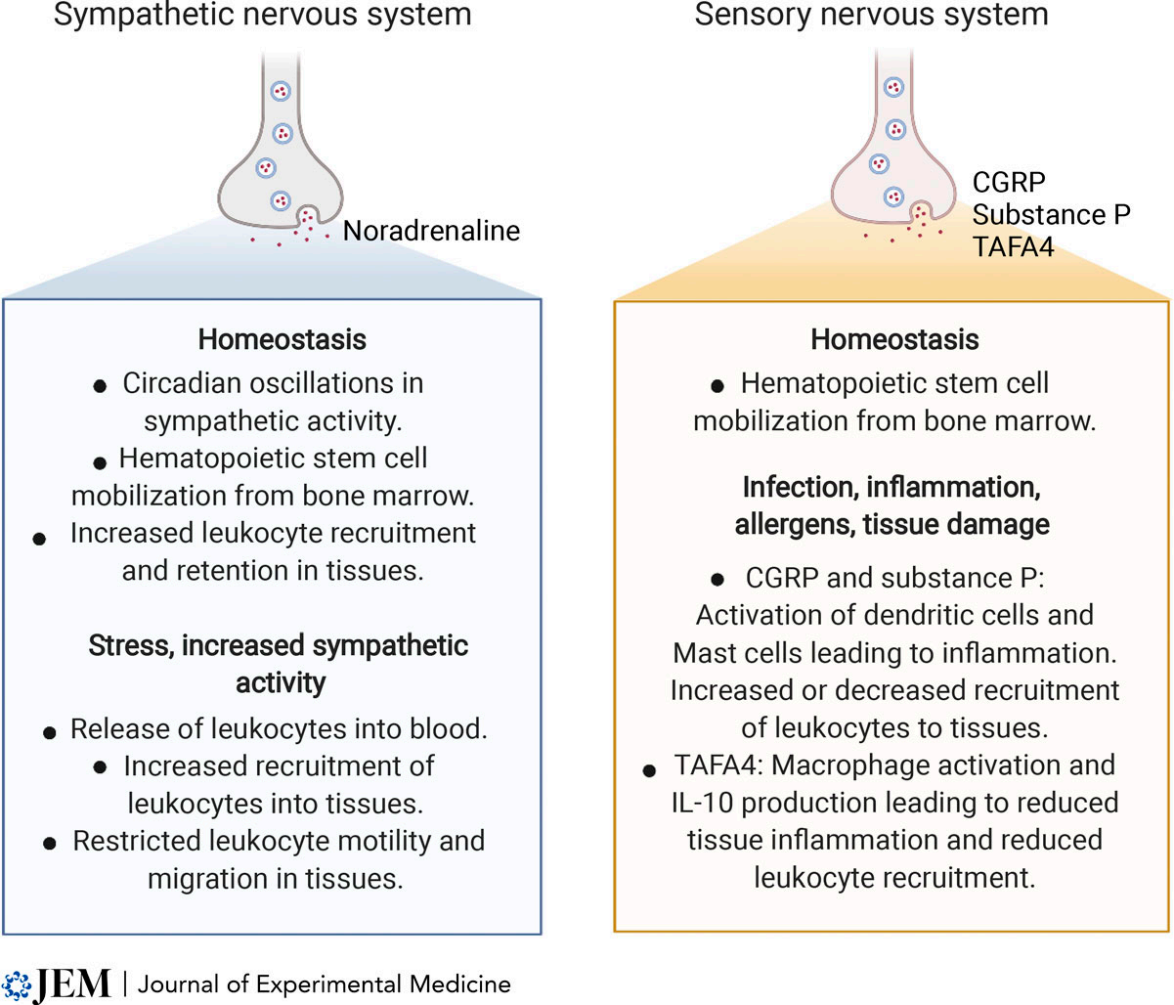
Summary of key sympathetic and sensory neural pathways and neurotransmitters that influence leukocyte trafficking and migration during homeostasis and during stress, tissue damage, or disease.

Neural signals influence immune responses by guiding leukocyte functions, including the migration of cells around the body and within tissues. In particular, catecholamines direct circadian oscillations in immune cell trafficking and can impair dynamic leukocyte motility in tissues (Fig. 4). There is widespread clinical use of catecholamines and adrenergic agonists as vasopressors to treat diseases including heart failure and hypotension caused by sepsis, as well as medications for asthma and allergic reactions. It will be important to better define the impact of these treatments on leukocyte trafficking and immune responses in patients in response to treatment. Conversely, the prevalent use of β-blockers to treat high blood pressure and cardiovascular diseases has the propensity to alter circadian regulation of leukocyte trafficking and also to reduce the positive influence of adrenergic signaling on the generation of T and B cell responses. Examples of sensory nerve activation contributing to pro- or antiinflammatory immune reactions and influencing the recruitment of leukocytes into damaged or infected tissues (Fig. 4) suggest that new treatment opportunities also exist for targeting sensory nerves. Still, we have much to learn about how peripheral neural signals influence immune cell migration and functions within healthy tissues and in tumors, and further research is needed if we are to design rational and safe treatments that target neuro-immune interactions.
